# Predicting recurrence risk after neoadjuvant therapy in breast cancer: a single-center retrospective cohort study integrating residual cancer burden and immune microenvironment features

**DOI:** 10.3389/fonc.2026.1925326

**Published:** 2026-10-14

**Authors:** Zhihua Zhao, Rui Deng, Liangliang Bai, Xiaogang Duan, Jingyuan Yi

**Affiliations:** 1 Physical Examination Center, Wang Jiang Hospital, Sichuan University, Chengdu, Sichuan, China; 2 Cancer Center, West China Hospital, Sichuan University, Chengdu, Sichuan, China; 3 Pharmacy Department, Chengdu Eighth People’s Hospital, Chengdu, Sichuan, China; 4 General Inpatient Department, West China Health Care Hospital, Sichuan University, Chengdu, Sichuan, China

**Keywords:** breast cancer, immune microenvironment, neoadjuvant therapy, residual cancer burden, tumor-infiltrating lymphocytes

## Abstract

**Background:**

Residual cancer burden (RCB) after neoadjuvant therapy (NAT) provides prognostic information, but its combination with immune microenvironment features for recurrence prediction remains underexplored.

**Methods:**

This single-center retrospective cohort study included 420 consecutive patients with breast cancer who underwent neoadjuvant therapy followed by definitive surgery between January 2015 and June 2024. Residual cancer burden (RCB) and post-neoadjuvant immune features, including tumor-infiltrating lymphocytes (TILs), CD8, FOXP3, and the proportion of CD163-positive among CD68-positive macrophages, were assessed. Patients were stratified by calendar era and randomly partitioned within strata into a training cohort (n=294) and an internal hold-out validation cohort (n=126). A LASSO-penalized Cox workflow was used for predictor selection, followed by unpenalized Cox refitting. Model performance was evaluated using C-statistics, time-specific Brier scores, calibration, bootstrap internal validation, and 5-year time-dependent decision curve analysis.

**Results:**

During a median follow-up of 61 months, 126 first recurrence events were observed (88 training and 38 validation). The final combined model retained RCB category, triple-negative breast cancer (TNBC), CD8 status, and CD163-positive macrophage proportion. The combined model achieved C-statistics of 0.79 (95% CI 0.75-0.83) in the training cohort and 0.75 (95% CI 0.69-0.81) in the internal validation cohort, with corresponding 5-year Brier scores of 0.142 and 0.158. Bootstrap internal validation yielded an optimism-corrected C-statistic of 0.772 and calibration slope of 0.92. The combined model provided the greatest net benefit among the evaluated models over 5-year threshold probabilities of approximately 15%-45%.

**Conclusions:**

Integration of RCB with TNBC status and post-neoadjuvant CD8 and CD163-positive macrophage features may provide incremental prognostic information for recurrence after neoadjuvant therapy. The model showed acceptable internal discrimination and calibration, but external validation is required before clinical implementation or treatment-directed use.

## Introduction

Breast cancer remains the most commonly diagnosed malignancy and leading cause of cancer-related mortality among women worldwide. Neoadjuvant therapy (NAT) has become an established treatment approach for locally advanced and high-risk early breast cancer, offering several advantages including tumor downstaging, facilitation of breast-conserving surgery, and provision of *in vivo* assessment of treatment sensitivity (1). The evaluation of pathological response to NAT has emerged as a crucial prognostic indicator that can guide subsequent therapeutic decisions (2).

The concept of pathological complete response (pCR), defined as the absence of invasive cancer in the breast and lymph nodes, has been widely adopted as a surrogate endpoint in neoadjuvant clinical trials and is associated with improved long-term outcomes, particularly in aggressive tumor subtypes such as triple-negative breast cancer (TNBC) and HER2-positive disease (3). However, pCR represents a binary classification that occurs in only approximately 20-30% of patients, leaving the majority with residual disease requiring more granular prognostic assessment (4). Additionally, the prognostic value of pCR varies considerably across molecular subtypes, being strongest in TNBC and HER2-positive tumors but less pronounced in hormone receptor-positive/HER2-negative disease (5).

To address these limitations, the Residual Cancer Burden (RCB) scoring system was developed at MD Anderson Cancer Center as a continuous quantitative measure incorporating the dimensions of the residual tumor bed, cancer cellularity, number of positive lymph nodes, and size of the largest nodal metastasis (6). The RCB index categorizes patients into four classes (RCB-0 to RCB-III), providing refined prognostic stratification that correlates with long-term survival outcomes across breast cancer subtypes (7). Recent multicenter validation studies have demonstrated the reproducibility and clinical utility of RCB assessment, establishing it as a valuable prognostic tool in the post-neoadjuvant setting (8).

Parallel to advances in pathological response assessment, accumulating evidence has highlighted the critical role of the tumor immune microenvironment in determining treatment outcomes and disease progression (9). Tumor-infiltrating lymphocytes (TILs), evaluated on standard hematoxylin and eosin-stained sections, have emerged as an accessible and reproducible biomarker with both predictive and prognostic significance in breast cancer (10). Multiple large-scale studies have demonstrated that high pre-treatment TILs are associated with increased rates of pCR to neoadjuvant chemotherapy, particularly in TNBC and HER2-positive subtypes (11). Furthermore, elevated TILs have been linked to improved disease-free survival and overall survival in these aggressive tumor types, supporting the hypothesis that breast cancer can be immunogenic and potentially responsive to immune-modulating therapies (12, 13).

Beyond overall lymphocytic infiltration, characterization of specific immune-cell populations provides additional information about the post-treatment tumor microenvironment. CD8+ cytotoxic T lymphocytes represent key effector cells capable of targeting tumor cells, whereas FOXP3+ regulatory T cells and macrophage phenotypes associated with immunoregulatory activity may attenuate antitumor immunity (14, 15). CD163 is commonly used to characterize a macrophage phenotype enriched in alternatively activated states; however, CD163 positivity alone does not establish fixed M2 polarization. Accordingly, the present study treats CD163 as a quantitative macrophage phenotypic marker rather than as a definitive surrogate for M2 identity.

Despite the established prognostic value of both RCB and immune biomarkers individually, integrated models combining these complementary sources of information remain scarce. Most previous studies have examined RCB or immune parameters in isolation, and comprehensive analyses incorporating multidimensional immune features alongside pathological response assessment are limited. The development of such integrated prediction models could potentially enhance risk stratification beyond what is achievable with traditional clinicopathological variables alone, enabling more personalized selection of adjuvant treatment strategies for patients with residual disease after NAT.

This study aimed to develop and internally validate a prognostic model integrating RCB classification with post-neoadjuvant immune features for prediction of recurrence after definitive breast surgery. The prespecified immune measurements included TILs, CD8+ cell density, FOXP3+ cell density, and the proportion of CD163-positive cells among CD68-positive macrophages. We further examined whether immune-only phenotypes constructed without RCB were associated with residual disease burden and whether selected immune features provided incremental prognostic information beyond RCB.

## Methods

### Study design and ethical considerations

#### Study type and temporal scope

This investigation was conducted as a single-center retrospective cohort study. Eligible patients were identified from consecutive cases of primary breast cancer treated with neoadjuvant therapy followed by definitive breast surgery at our institution between January 2015 and June 2024. The primary prognostic factors of interest were RCB in post-surgical specimens and quantitatively assessed post-treatment immune features. Clinical, pathological, treatment, and follow-up information was retrospectively collected from the hospital medical-record system and institutional follow-up database.

#### Ethical approval and informed consent

The Institutional Research Ethics Committee reviewed the study and determined that formal ethical approval was not required because this retrospective investigation used existing de-identified clinical and pathological data. The requirement for individual informed consent was waived. All procedures were conducted in accordance with the Declaration of Helsinki and institutional requirements for protection of patient confidentiality.

#### Overall study framework

The comprehensive study workflow encompassed the following sequential steps: (1) systematic screening and enrollment of eligible cases; (2) extraction of clinical and treatment-related information from medical records; (3) calculation of RCB scores from post-surgical pathological specimens; (4) quantitative assessment and characterization of immune microenvironment indicators; (5) ascertainment and confirmation of recurrence events and survival outcomes through structured follow-up; (6) development of statistical prediction models and evaluation of their performance metrics. A detailed patient flow diagram illustrating the progression from initial screening through final cohort formation is presented in the Results section.

### Study participants

#### Inclusion criteria

Patients were eligible if they had histologically confirmed invasive breast carcinoma, received neoadjuvant therapy followed by definitive breast surgery at our institution during the study period, had pre-treatment imaging and molecular subtype information sufficient for clinicopathological characterization, had surgical specimens adequate for RCB assessment and post-treatment immune-marker evaluation, and had at least one valid postoperative disease assessment. A minimum follow-up duration was not required for eligibility; patients with shorter follow-up were retained and censored at their last evaluable disease assessment.

#### Exclusion criteria

Patients were excluded for distant metastatic disease at initial presentation, another malignancy within the preceding 5 years that could materially affect outcome ascertainment, absence of critical clinicopathological information required for RCB calculation or recurrence assessment, absence of any evaluable postoperative follow-up information, or premature discontinuation of neoadjuvant therapy for reasons that precluded standardized response assessment. Short follow-up duration alone was not an exclusion criterion. Two investigators applied eligibility criteria independently, with discrepancies resolved by consensus with a senior investigator.

#### Sample size estimation and cohort partitioning

This retrospective consecutive-case cohort included all eligible patients treated during the defined study period rather than a sample expanded to achieve a prespecified number of events. Patients were stratified according to calendar era (2015–2019 vs 2020-June 2024) and randomly partitioned within each stratum at an approximately 7:3 ratio using a computer-generated random sequence. This yielded 294 patients in the training cohort and 126 in the internal hold-out validation cohort, with both calendar periods represented in each cohort. The training cohort contained 88 recurrence events. Given the available event count, model development used a parsimonious penalized-selection strategy; the final model contained six effective degrees of freedom, and subgroup analyses were treated as exploratory.

### Neoadjuvant treatment protocols and surgical management

#### Neoadjuvant treatment regimens

Neoadjuvant therapy regimens were determined through multidisciplinary team consensus, incorporating tumor characteristics and molecular subtype information. For HER2-positive disease, treatment primarily consisted of combination chemotherapy with targeted therapy (trastuzumab with or without pertuzumab). Triple-negative breast cancer patients predominantly received anthracycline-taxane sequential chemotherapy, with select cases receiving additional immunotherapy with checkpoint inhibitors. Hormone receptor-positive/HER2-negative tumors were treated with chemotherapy regimens, with intensity and duration adjusted according to risk stratification. All medication dosing and cycle numbers adhered to contemporary international guidelines and institutional standards, with actual treatment combinations subsequently categorized into distinct regimen subgroups for analytical purposes.

#### Surgical approach and axillary management

Following completion of NAT, patients underwent either breast-conserving surgery or modified radical mastectomy based on clinical response assessment through imaging and multidisciplinary team evaluation. Axillary management incorporated sentinel lymph node biopsy (SLNB) and/or axillary lymph node dissection (ALND), with the specific approach determined by pre- and post-NAT axillary nodal status according to guideline recommendations. Patients with negative SLNB generally did not proceed to completion ALND, whereas those with positive sentinel nodes underwent individualized decision-making regarding the need for additional axillary clearance based on tumor burden and clinical factors.

#### Adjuvant treatment and follow-up strategy

Postoperative treatment was determined by multidisciplinary assessment of molecular subtype, pathological stage, RCB, treatment response, and contemporaneous clinical standards. Across the full cohort, 46 patients (11.0%) received capecitabine, 143 (34.0%) postoperative HER2-directed therapy, 244 (58.1%) endocrine therapy, 326 (77.6%) radiotherapy, and 41 (9.8%) continued immunotherapy. Treatment-era effects were considered because 181 patients underwent surgery during 2015–2019 and 239 during 2020-June 2024. Follow-up evaluations were scheduled every 3–6 months during the first 2 postoperative years, every 6–12 months during years 3-5, and annually thereafter, with recurrence and vital status documented from clinical visits, imaging, pathology, and structured follow-up contact.

### Specimen processing and pathological evaluation

#### Specimen handling

All surgical specimens were delivered to the Department of Pathology within 30 minutes of excision and fixed in 10% neutral buffered formalin for 24–48 hours, followed by standard dehydration and paraffin embedding procedures. The breast tumor bed was serially sectioned perpendicular to the maximum diameter, with measurement and documentation of the extent of residual tumor. Lymph nodes were carefully dissected, with total number and number of metastatic nodes recorded, along with measurement of the largest metastatic focus diameter. Representative sections were cut at 4 μm thickness for hematoxylin and eosin staining, immunohistochemistry, and multiplex immunofluorescence studies as indicated.

#### Molecular subtyping and conventional pathological response assessment

Molecular subtype classification followed American Society of Clinical Oncology/College of American Pathologists guidelines for immunohistochemical assessment of estrogen receptor, progesterone receptor, HER2, and Ki-67, defining Luminal A, Luminal B (HER2-negative and HER2-positive), HER2-enriched, and triple-negative subtypes. Both pre-treatment core biopsies and surgical specimens were evaluated for subtype confirmation. Conventional pathological response assessment utilized the Miller-Payne grading system and other established classification schemes to evaluate NAT efficacy, providing comparative data for analysis alongside RCB stratification.

### Residual cancer burden scoring

#### RCB calculation

The RCB index was calculated from the definitive post-neoadjuvant surgical specimen using the standard components: two dimensions of the residual primary tumor bed, overall cancer cellularity, the percentage of *in situ* disease within the tumor bed used to derive invasive cellularity, the number of positive lymph nodes, and the diameter of the largest nodal metastasis. Two experienced breast pathologists independently evaluated the required components while blinded to outcome. Cases were referred to a third pathologist when the two reviewers assigned different RCB classes or when the absolute difference in continuous RCB index exceeded 0.50. The final RCB index and class were established by consensus and calculated using the established RCB algorithm.

#### RCB classification and inter-observer agreement

Patients were classified as RCB-0, RCB-I, RCB-II, or RCB-III using established RCB thresholds. Interobserver reproducibility was evaluated before consensus adjudication. The prognostic associations of RCB categories with recurrence were assessed as four-level categorical effects, with RCB-0 serving as the reference category in the final prediction model.

### Immune microenvironment feature assessment

#### Tumor-infiltrating lymphocytes evaluation

Stromal TILs were assessed on post-neoadjuvant surgical specimens according to International TILs Working Group principles (10). Evaluation focused on the histologically identified post-treatment tumor bed and associated stroma. In RCB-0 cases, in which no residual invasive carcinoma was present, the previously identified treated tumor-bed/stromal area was used rather than a residual invasive-tumor compartment. TILs were recorded as a continuous percentage. Two pathologists independently reviewed each case, and absolute scoring differences greater than 10 percentage points triggered joint re-review. The interobserver intraclass correlation coefficient for TILs was 0.89 (95% CI 0.86-0.91).

#### Immunohistochemical detection of CD8, FOXP3, CD68, and CD163

CD8, FOXP3, CD68, and CD163 were evaluated on post-neoadjuvant surgical specimens using the pathology laboratory’s validated clinical immunohistochemical assays. Whole-slide images were reviewed at up to x40 magnification. Five representative regions of interest within the residual tumor bed/stromal compartment were selected for each case; in RCB-0 cases, the histologically identified treated tumor-bed/stromal area was used. CD8+ and FOXP3+ cells were quantified as cells/mm². The macrophage variable was defined as CD163-positive macrophages divided by CD68-positive macrophages x100% and is referred to as the CD163-positive macrophage proportion rather than as a definitive M2 measurement. Two pathologists independently reviewed the measurements, with differences greater than 10% undergoing joint adjudication. Interobserver ICCs were 0.93 (95% CI 0.91-0.95) for CD8, 0.88 (95% CI 0.85-0.91) for FOXP3, and 0.90 (95% CI 0.87-0.92) for CD163 proportion. Archived assay metadata did not permit uniform reconstruction of antibody clone and manufacturer information for every case; therefore, no unverified reagent identifiers were assigned.

#### Construction of comprehensive immune features and immune microenvironment subtypes

Immune phenotypes were constructed exclusively from TILs, CD8+ cell density, FOXP3+ cell density, and CD163-positive macrophage proportion. The continuous immune variables were standardized before unsupervised hierarchical clustering, and a three-cluster solution was used to summarize immune-feature patterns as immune-active, immune-intermediate, and immune-suppressed phenotypes. RCB was not used to construct the clusters. Principal component analysis was likewise performed using only the four immune variables. RCB groups were overlaid only after the immune feature space had been generated, thereby permitting evaluation of the relationship between immune contexture and residual disease without incorporating RCB into the dimension-reduction procedure.

### Follow-up and endpoint definitions

#### Follow-up methodology

Follow-up began on the date of definitive surgery and continued until first documented recurrence, death, last evaluable disease assessment, or study closure. Local-regional and distant recurrences were confirmed using radiological, pathological, or multidisciplinary clinical evidence. Thirty-one patients had less than 24 months of follow-up but had valid postoperative outcome assessment and were retained in the time-to-event analysis with censoring at their last evaluable assessment. Follow-up data underwent structured quality review before outcome analysis.

#### Outcome definitions

The primary endpoint was recurrence-free interval (RFI), operationally defined as the time from definitive surgery to the first documented locoregional or distant recurrence. Patients without recurrence were censored at the date of their last disease assessment. Death without a previously documented recurrence was treated as a censoring event at the date of death in the primary analysis. A sensitivity analysis used a composite recurrence-free survival endpoint in which death without prior recurrence was additionally counted as an event. Distant recurrence was also summarized separately as a descriptive outcome.

### Statistical analysis

#### Data preprocessing

Continuous variables are summarized using mean and standard deviation or median and interquartile range, as appropriate, and categorical variables using frequencies and percentages. The training and validation cohorts were defined before handling missing predictor data. Missing predictors were then imputed separately within the two cohorts using multiple imputation by chained equations with 20 imputed datasets; no RCB or outcome data were missing. In total, 23 patients (5.5%) had at least one missing candidate predictor, including 16/294 (5.4%) in training and 7/126 (5.6%) in validation. Model development, preprocessing, cutoff determination, and predictor selection were restricted to the training cohort. Final regression estimates from imputed training datasets were combined using standard multiple-imputation pooling, and complete-case analysis was performed as a sensitivity analysis.

#### Univariable and multivariable Cox regression

Candidate predictors were evaluated within the training cohort using LASSO-penalized Cox regression. Twelve candidate predictor groups were entered into the penalized modeling workflow. Ten-fold cross-validation was used to select the penalty parameter using the one-standard-error rule (lambda1se=0.031). The retained predictor groups were RCB, molecular subtype represented by TNBC status, CD8, and CD163-positive macrophage proportion. These predictors were then refitted in an unpenalized Cox model to obtain interpretable coefficients, hazard ratios, and 95% confidence intervals. Univariable associations were summarized descriptively and were not used as a significance-based gate for LASSO eligibility. The proportional-hazards assumption was assessed using Schoenfeld residuals.

#### Combined risk score and nomogram construction

The final linear predictor was LP = 0.278I(RCB-I) + 0.770I(RCB-II) + 1.399I(RCB-III) + 0.392I(TNBC) - 0.446I(CD8-high) + 0.470I(CD163-high), with all indicators coded 0/1 and RCB-0 as the reference. Training-derived dichotomization thresholds were locked before validation: CD8-high was defined as >=315 cells/mm² and CD163-high as >=58% of CD68-positive macrophages. Baseline RFI estimates from the training Cox model were S0(3 years)=0.932 and S0(5 years)=0.875, yielding individual predictions as S(t|X)=S0(t)^exp(LP). Training-cohort tertiles of the linear predictor defined low-, intermediate-, and high-risk groups using cutoffs of 0.42 and 1.08; these cutoffs were applied unchanged to the validation cohort.

#### Model performance evaluation and internal validation

Model discrimination was quantified using Harrell’s C-statistic with 95% confidence intervals. Prediction error was summarized using time-specific Brier scores at 3 and 5 years, reported separately for training and validation cohorts. Calibration was evaluated numerically using time-specific calibration slopes and intercepts and graphically in the internal hold-out validation cohort. Bootstrap internal validation with 1,000 resamples was performed in the training cohort; the complete model-development procedure, including preprocessing, cutoff determination, LASSO selection, Cox refitting, and performance calculation, was repeated in each resample to estimate optimism. Time-dependent decision curve analysis was performed for 5-year recurrence prediction in the internal validation cohort using survival-based net-benefit estimation that accounted for censoring. Clinical, RCB-only, immune-only, and combined models were compared with treat-all and treat-none strategies.

#### Subgroup and sensitivity analyses

Molecular-subtype subgroup analyses were considered exploratory because of limited event counts within individual strata. The training-derived risk-group thresholds were applied unchanged, and interaction terms were used to assess heterogeneity. Sensitivity analyses included a treatment-adjusted model incorporating postoperative capecitabine, HER2-directed therapy, endocrine therapy, radiotherapy, continued immunotherapy, and calendar era; exclusion of RCB-0 cases to evaluate immune-feature assessment in non-pCR disease; complete-case analysis; and the composite endpoint that counted death without prior recurrence as an event.

## Results

### Patient baseline characteristics and follow-up information

A total of 512 patients were screened. Fifty-four were excluded before detailed eligibility assessment because of distant metastasis at presentation (n=22), another malignancy within the preceding 5 years (n=18), or incomplete critical baseline/pathological data (n=14). Among 458 otherwise eligible patients, 24 had no evaluable postoperative follow-up information and 14 had prematurely discontinued neoadjuvant treatment in a manner that precluded standardized response assessment. The final cohort therefore comprised 420 patients. Calendar-era-stratified random partitioning generated a training cohort of 294 patients and an internal hold-out validation cohort of 126 patients (**Figure 1**).

**Figure 1:**
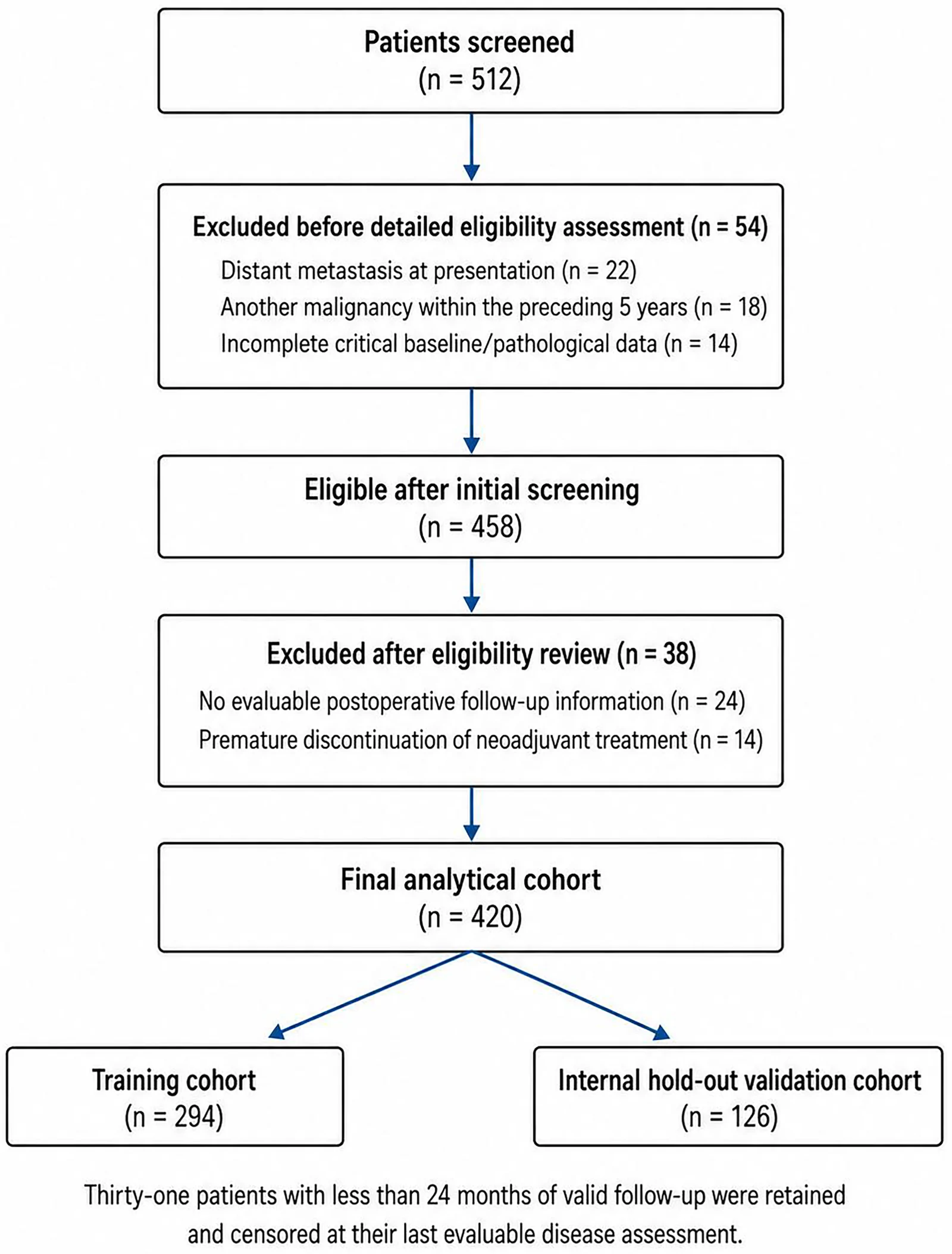
Patient screening and cohort construction flow diagram. The flowchart shows screening of 512 patients, exclusion of cases without required eligibility or evaluable postoperative outcome information, and the final cohort of 420 patients. The cohort was partitioned within calendar-era strata into a training cohort (n=294) and an internal hold-out validation cohort (n=126). Thirty-one patients with less than 24 months of valid follow-up were retained and censored normally.

Baseline characteristics are summarized in **Table 1**. Median follow-up was 61 months overall, 62 months in training, and 60 months in validation. During follow-up, 126 first recurrences occurred, including 31 locoregional and 95 distant recurrences; 88 events occurred in training and 38 in validation. Nine patients died without a previously documented recurrence (6 training and 3 validation) and were censored at death in the primary RFI analysis. The proportions treated during 2015–2019 were 42.9% in training and 43.7% in validation, with both calendar eras represented in each cohort. Postoperative treatment distributions were broadly similar between cohorts and are detailed in **Supplementary Table S1**.

**Table 1:** Baseline clinicopathological characteristics, calendar era, follow-up, and outcome events.

Characteristic	Overall (n=420)	Training set (n=294)	Validation set (n=126)	P Value
Age, median (IQR), years	50 (43–59)	50 (43–59)	51 (44–60)	0.62
Menopausal status (Post/Pre)	260/160	181/113	79/47	0.95
Clinical T stage (T1/T2/T3–4)	60/270/90	42/190/62	18/80/28	0.88
Clinical N stage (N0/N1–3)	130/290	92/202	38/88	0.79
Molecular subtype				
Luminal A	76 (18.1%)	54 (18.4%)	22 (17.5%)	0.97
Luminal B	178 (42.4%)	124 (42.2%)	54 (42.9%)	
HER2-enriched	92 (21.9%)	64 (21.8%)	28 (22.2%)	
Triple-negative	74 (17.6%)	52 (17.7%)	22 (17.5%)	
Surgery type (BCS/Mastectomy)	190/230	132/162	58/68	0.88
Follow-up time, median (months)	61 (46–82)	62 (47–83)	60 (45–80)	0.54
Surgery era (2015-2019 / 2020-Jun 2024)	181 / 239	126 / 168	55 / 71	0.97
Recurrence events, n (%)	126 (30.0%)	88 (29.9%)	38 (30.2%)	–
Death without prior recurrence, n	9	6	3	–

### RCB classification and conventional pathological response assessment

Post-neoadjuvant RCB classification revealed that 119 patients (28.3%) achieved pathological complete response (RCB-0), while 64 patients (15.2%) had minimal residual disease (RCB-I), 162 patients (38.6%) had moderate residual disease (RCB-II), and 75 patients (17.9%) had extensive residual disease (RCB-III) as shown in **Table 2**. The distribution of RCB categories differed significantly across molecular subtypes (P<0.001). Triple-negative and HER2-enriched tumors demonstrated higher pCR rates (43% and 38% respectively), whereas Luminal subtypes predominantly had RCB-II or RCB-III disease, reflecting differential chemosensitivity patterns.

**Table 2:** Neoadjuvant and postoperative treatment characteristics.

Variable	n (%)
NAT Regimen	
Anthracycline + Taxane	220 (52.4%)
Anthracycline + Taxane + HER2-targeted	96 (22.9%)
Taxane + HER2-targeted	54 (12.9%)
Including immune checkpoint inhibitor	50 (11.9%)
NAT cycles, median (IQR)	6 (6–8)
Conventional pathological response	
Complete response (pCR)	119 (28.3%)
Partial response	213 (50.7%)
Stable/Progressive disease	88 (21.0%)
Postoperative treatment	
Capecitabine	46 (11.0%)
HER2-directed therapy	143 (34.0%)
Endocrine therapy	244 (58.1%)
Radiotherapy	326 (77.6%)
Continued immunotherapy	41 (9.8%)

Comparison between RCB classification and conventional Miller-Payne pathological response grading revealed moderate concordance, with a weighted kappa coefficient of 0.62 (95% CI: 0.56-0.68) as presented in **Table 3**. While both systems demonstrated prognostic value, RCB provided more granular stratification of recurrence risk. The 5-year recurrence rates increased progressively with higher RCB class: 8.4% for RCB-0, 12.5% for RCB-I, 24.7% for RCB-II, and 45.3% for RCB-III (log-rank P<0.001).

**Table 3:** RCB category distribution and concordance with conventional grading.

RCB category	n (%)	pCR (n)	Non-pCR (n)	5-Year recurrence rate (%)
RCB-0	119 (28.3%)	119	0	8.4
RCB-I	64 (15.2%)	0	64	12.5
RCB-II	162 (38.6%)	0	162	24.7
RCB-III	75 (17.9%)	0	75	45.3
Weighted Kappa value	—	—	—	0.62

**Figure 2** Sankey diagram of molecular subtype distribution across RCB categories. This alluvial plot visualizes the flow of 420 patients from four molecular subtypes to four RCB categories.

**Figure 2:**
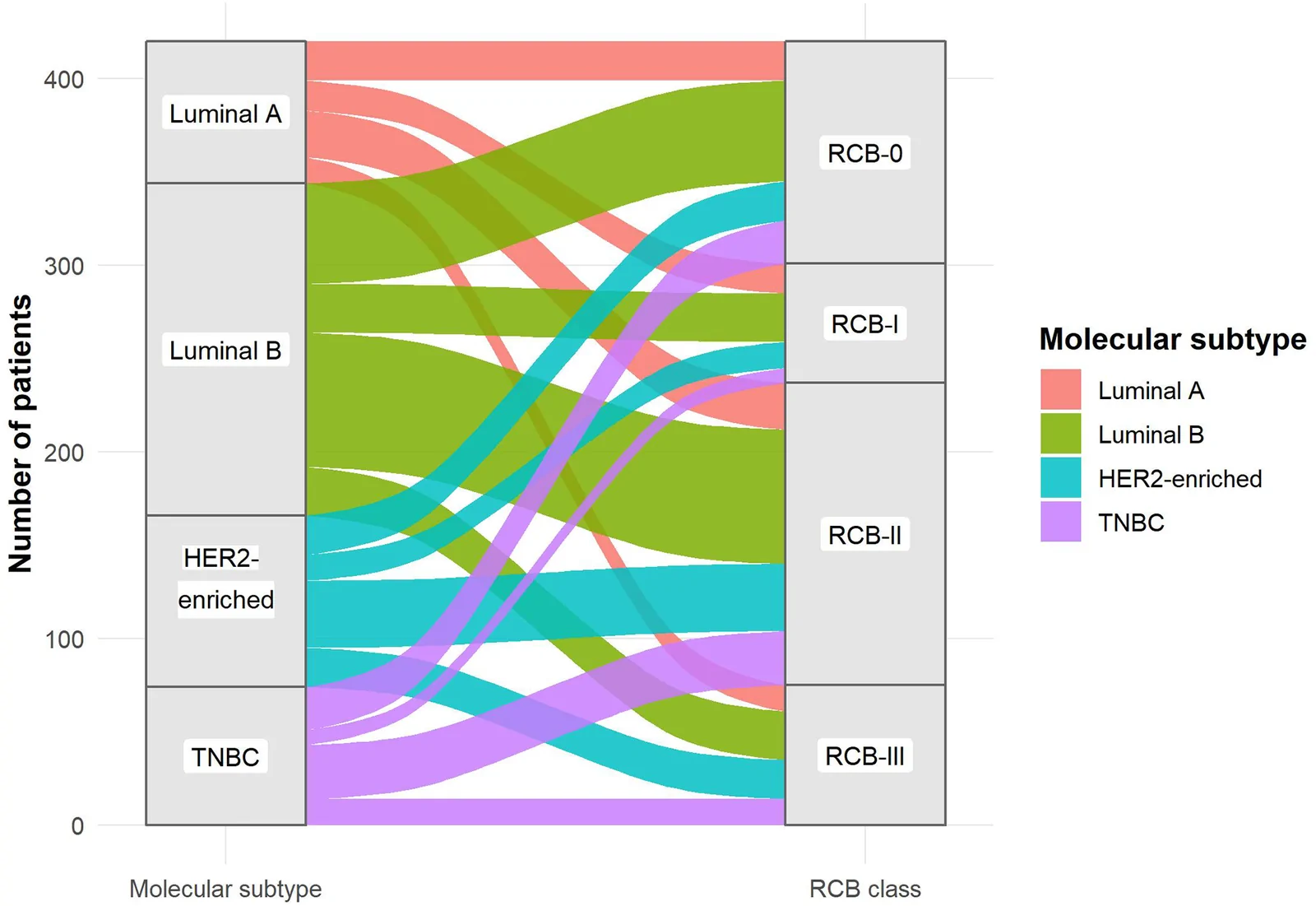
Sankey diagram of molecular subtype distribution across RCB categories. Alluvial plot showing the flow of 420 patients from four molecular subtypes (Luminal A, Luminal B, HER2-enriched, TNBC) on the left to four RCB categories (RCB-0, RCB-I, RCB-II, RCB-III) on the right. Flow width represents patient numbers. Triple-negative and HER2-enriched subtypes show higher proportions achieving RCB-0/I, while Luminal subtypes predominantly have RCB-II/III disease.

### Immune microenvironment feature distribution and clustering results

Evaluation of tumor-infiltrating lymphocytes revealed a median TILs level of 15% (IQR: 5-35%) across the entire cohort. Higher TILs were more frequently observed in triple-negative (median 25%) and HER2-enriched (median 22%) subtypes compared to hormone receptor-positive tumors (median 10%, P<0.001).

Post-treatment immune features varied substantially across patients. Median TILs were 15% (IQR 5%-35%), median CD8+ density was 320 cells/mm² (IQR 180-620), median FOXP3+ density was 85 cells/mm² (IQR 40-160), and median CD163-positive macrophage proportion was 58% (IQR 42%-72). CD8+ density was higher in RCB-0/I than in RCB-II/III disease, whereas FOXP3+ density and CD163-positive macrophage proportion were higher in the RCB-II/III group. These associations describe post-treatment immune contexture and do not imply a fixed macrophage polarization state.

**Figure 3** Correlation heatmap between RCB index and post-treatment immune markers. The matrix summarizes pairwise Pearson correlations among RCB, TILs, CD8, FOXP3, and CD163-positive macrophage proportion. The redundant M2-ratio variable was not used in the final immune-feature definition or prediction model.

**Figure 3:**
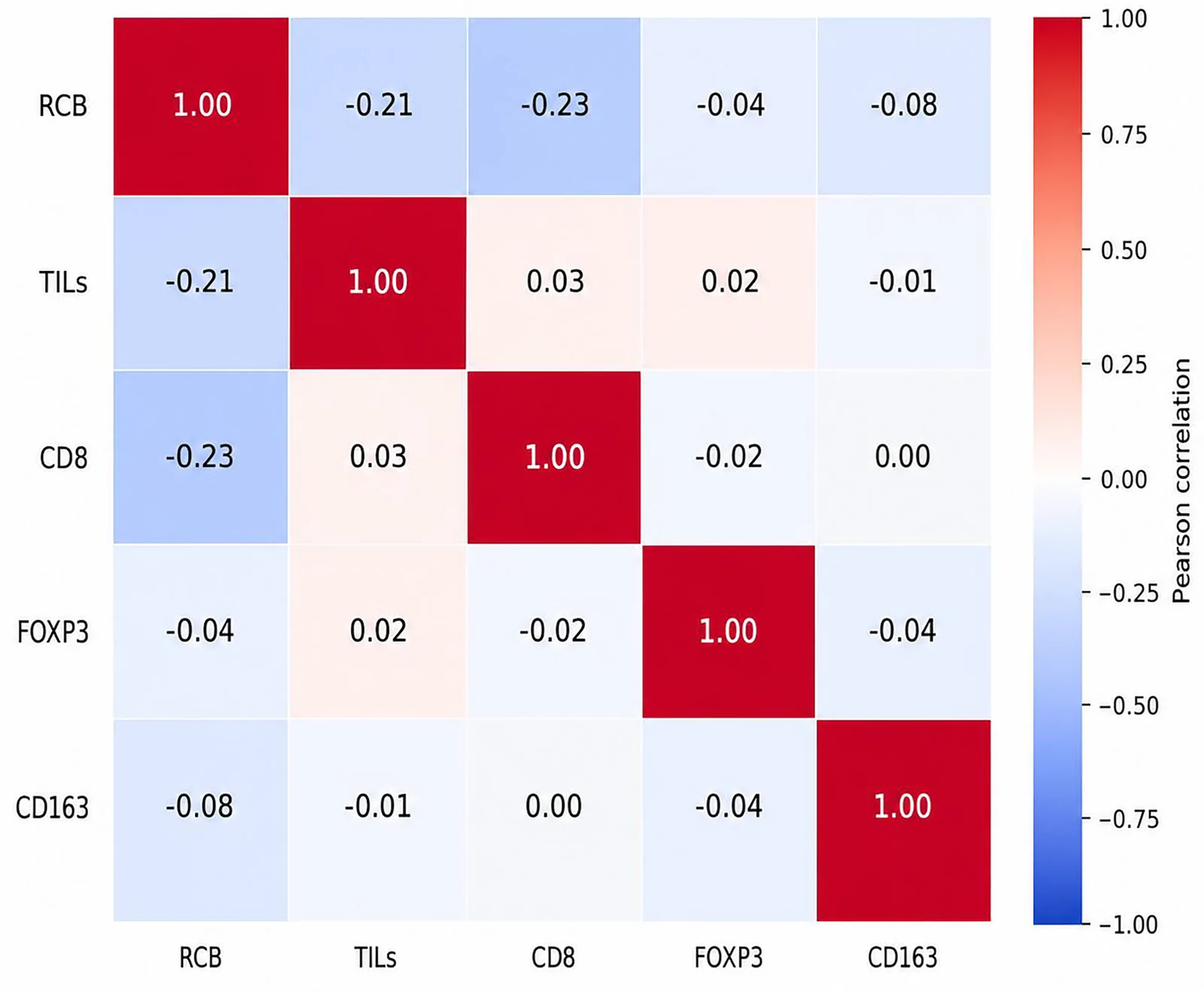
Correlation heatmap between RCB index and post-treatment immune markers. Pairwise Pearson correlations are shown for RCB, TILs, CD8, FOXP3, and CD163-positive macrophage proportion. The redundant M2-ratio terminology is not used.

**Figure 4** Immune-only clustering profile. Unsupervised clustering was constructed from TILs, CD8, FOXP3, and CD163-positive macrophage proportion only. The resulting immune-active (n=128), immune-intermediate (n=181), and immune-suppressed (n=111) groups showed stepwise differences in RCB distribution: RCB-0/I occurred in 53.9%, 43.6%, and 31.5%, respectively (P = 0.002) (see **Table 4**). RCB was overlaid after cluster construction and was not an input variable. 

**Figure 4:**
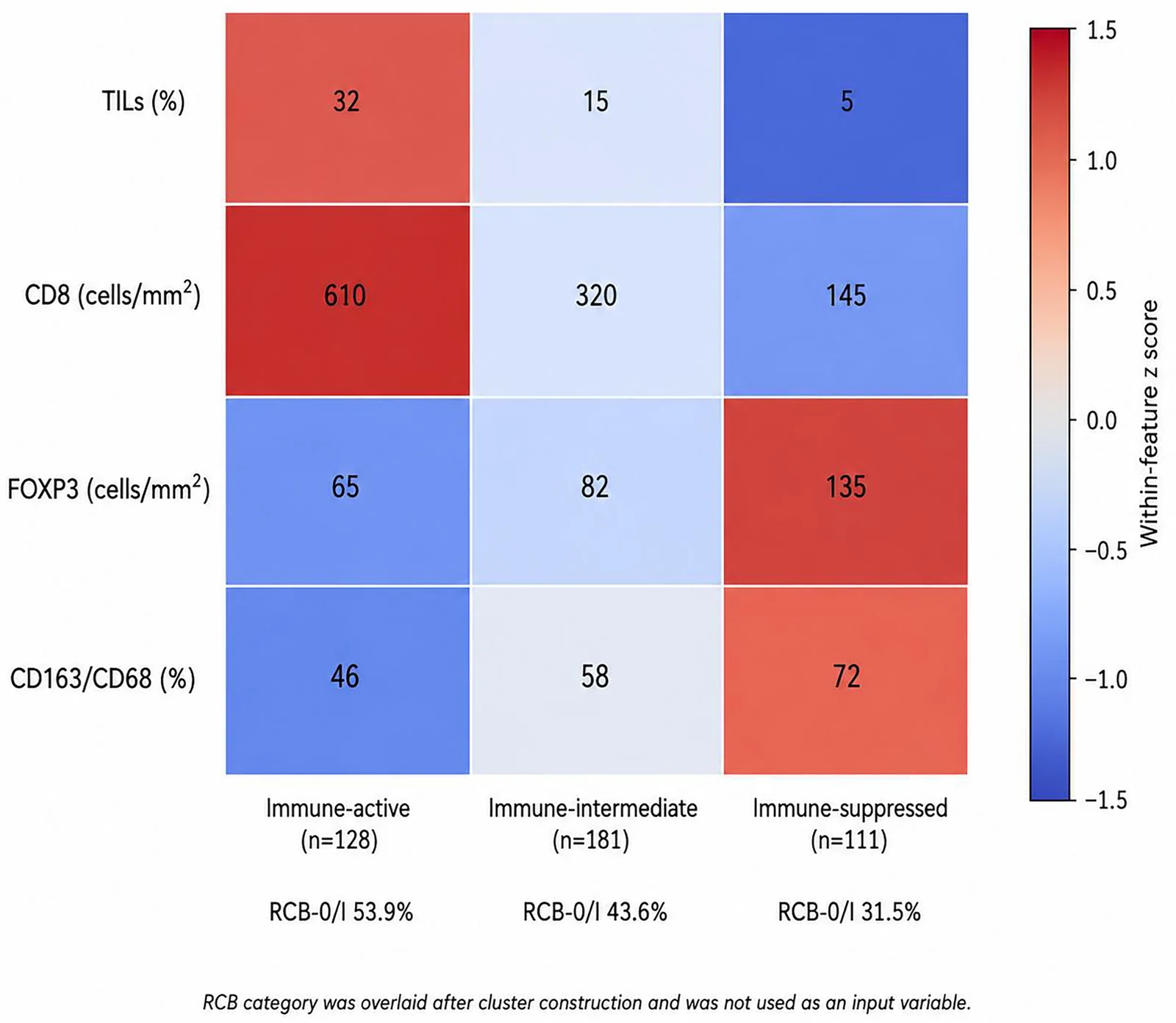
Immune-only clustering profile. Immune phenotypes were constructed from TILs, CD8, FOXP3, and CD163-positive macrophage proportion only. RCB was excluded from cluster construction and overlaid afterward. The three data-derived groups were labeled immune-active, immune-intermediate, and immune-suppressed; RCB-0/I frequencies were 53.9%, 43.6%, and 31.5%, respectively (P = 0.002).

**Table 4:** Immune feature profiles across data-derived immune phenotypes.

Immune feature	Immune-active n=128	Immune-intermediate n=181	Immune-suppressed n=111	P Value
TILs, %	32 (20-50)	15 (8-25)	5 (2-10)	<0.001
CD8+, cells/mm²	610 (420-840)	320 (220-450)	145 (90-230)	<0.001
FOXP3+, cells/mm²	65 (35-105)	82 (45-140)	135 (80-210)	<0.001
CD163+/CD68+, %	46 (35-58)	58 (45-70)	72 (62-81)	<0.001
RCB-0/I, n (%)	69 (53.9%)	79 (43.6%)	35 (31.5%)	0.002
RCB-II/III, n (%)	59 (46.1%)	102 (56.4%)	76 (68.5%)	

**Figure 5** Principal component analysis using only TILs, CD8, FOXP3, and CD163-positive macrophage proportion showed that PC1 explained 47.8% of variance and PC2 24.6%, for a cumulative 72.4%. PC1 loadings were +0.53 for TILs, +0.56 for CD8, -0.39 for FOXP3, and -0.50 for CD163 proportion. After PCA construction, RCB was overlaid as an external grouping variable. Mean PC1 score was 0.27 +/- 0.94 in RCB-0/I and -0.21 +/- 1.02 in RCB-II/III (P<0.001), supporting an association between residual burden and the independently constructed immune-feature axis.

**Figure 5:**
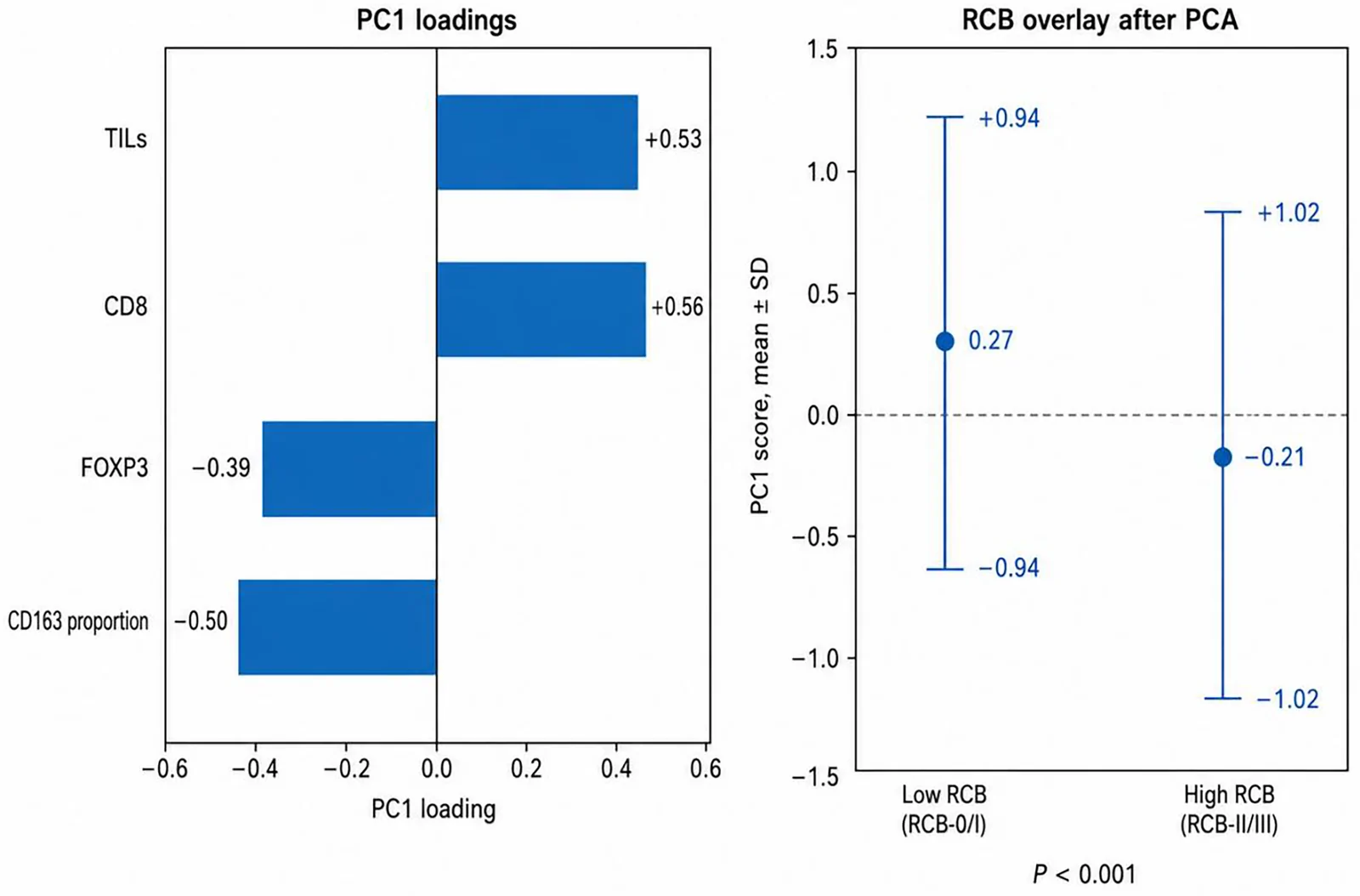
Principal component analysis summary based on immune features only. PCA used TILs, CD8, FOXP3, and CD163-positive macrophage proportion without RCB. PC1 and PC2 explained 47.8% and 24.6% of variance, respectively. PC1 loadings were +0.53 for TILs, +0.56 for CD8, -0.39 for FOXP3, and -0.50 for CD163 proportion. RCB was overlaid only after PCA; mean PC1 was 0.27 +/- 0.94 for RCB-0/I and -0.21 +/- 1.02 for RCB-II/III (P<0.001).

### Development of combined recurrence risk prediction model

The LASSO workflow in the training cohort retained four predictor groups: RCB, TNBC, CD8 status, and CD163-positive macrophage proportion. In the unpenalized Cox refit, RCB-II and RCB-III were associated with higher recurrence hazards than RCB-0, CD8-high status was associated with lower recurrence hazard, and CD163-high status with higher recurrence hazard (**Table 5**). TNBC remained in the prediction model despite a two-sided P value of 0.077 because predictor retention was determined by the penalized modeling procedure rather than by post-selection significance testing.

**Table 5:** Final multivariable Cox model for recurrence-free interval.

Predictor	beta coefficient	HR	95% CI	P value
RCB-0	Reference	1.00	–	–
RCB-I	0.278	1.32	0.61-2.86	0.48
RCB-II	0.770	2.16	1.15-4.06	0.016
RCB-III	1.399	4.05	2.08-7.89	<0.001
TNBC	0.392	1.48	0.96-2.28	0.077
CD8 high	-0.446	0.64	0.43-0.95	0.027
CD163 high	0.470	1.60	1.08-2.37	0.019

The final linear predictor was LP = 0.278I(RCB-I) + 0.770I(RCB-II) + 1.399I(RCB-III) + 0.392I(TNBC) - 0.446I(CD8-high) + 0.470I(CD163-high). Baseline RFI was 0.932 at 3 years and 0.875 at 5 years. For example, a patient with RCB-II, TNBC, CD8-low, and CD163-high had LP = 1.632, corresponding to an estimated 3-year RFI of approximately 69.9% and 5-year RFI of approximately 50.6%.

**Figure 6** Coefficient-based nomogram for recurrence-free interval. The nomogram represents the final Cox model incorporating four predictor groups: RCB category, TNBC status, CD8 status, and CD163-positive macrophage proportion. Individual 3- and 5-year RFI estimates are derived from the model linear predictor and training-cohort baseline survival.

**Figure 6:**
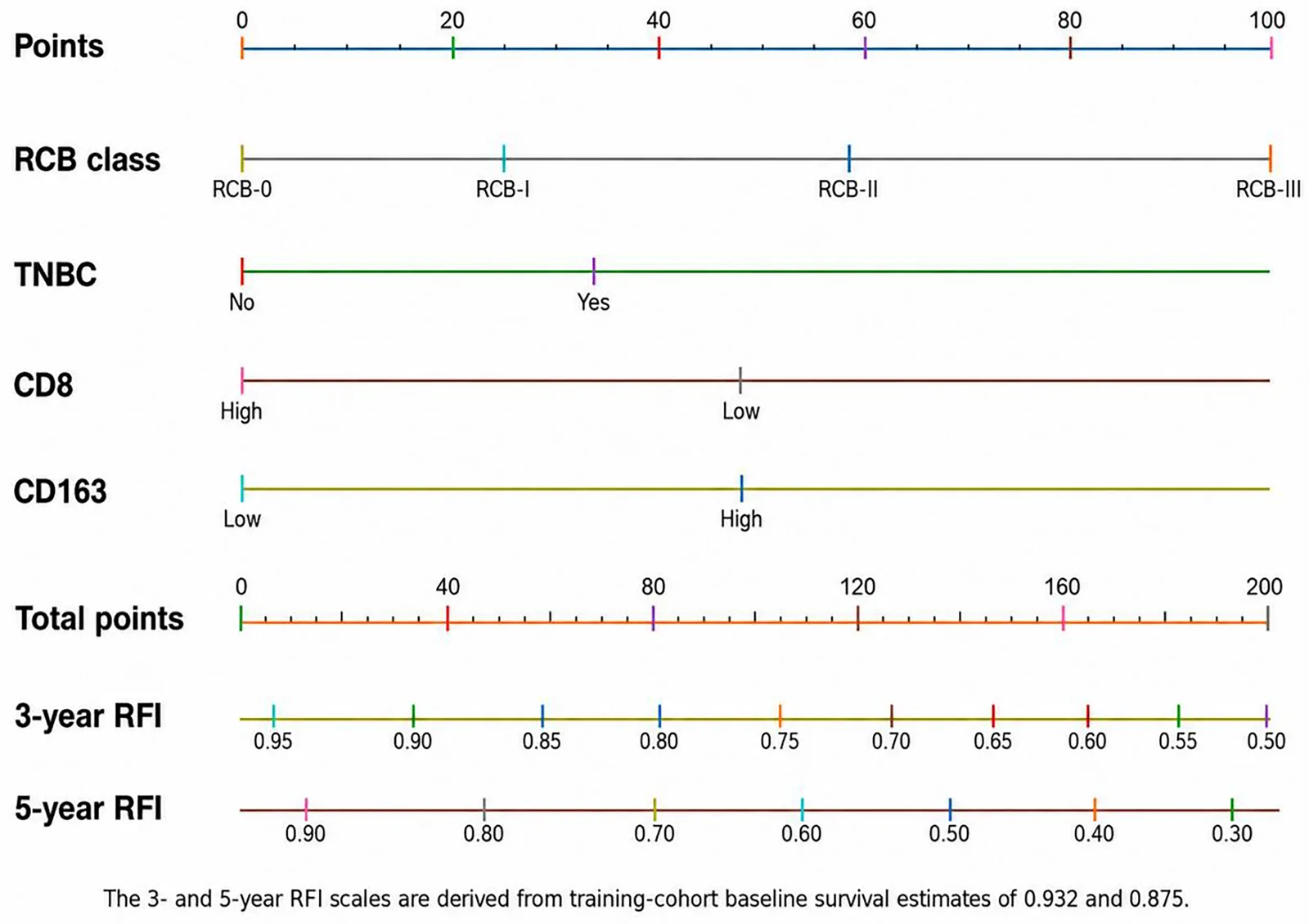
Coefficient-based nomogram for recurrence-free interval. The nomogram represents the final Cox model containing RCB category, TNBC status, CD8 status, and CD163-positive macrophage proportion. The 3- and 5-year RFI scales are derived from training-cohort baseline survival estimates of 0.932 and 0.875.

Training-cohort tertiles of the linear predictor yielded cutoffs of 0.42 and 1.08. In training, the low-, intermediate-, and high-risk groups each contained 98 patients and had 7 (7.1%), 23 (23.5%), and 58 (59.2%) recurrences, respectively. Applying the same locked cutoffs to validation yielded 45 low-risk, 38 intermediate-risk, and 43 high-risk patients with 4 (8.9%), 11 (28.9%), and 23 (53.5%) recurrences. Five-year Kaplan-Meier RFI estimates were 91.5%, 77.8%, and 47.1% in training and 88.9%, 74.9%, and 50.5% in validation (log-rank P<0.001 in each cohort) (see **Table 6**). Corresponding Kaplan-Meier curves are shown in **Figure 7**.

**Figure 7:**
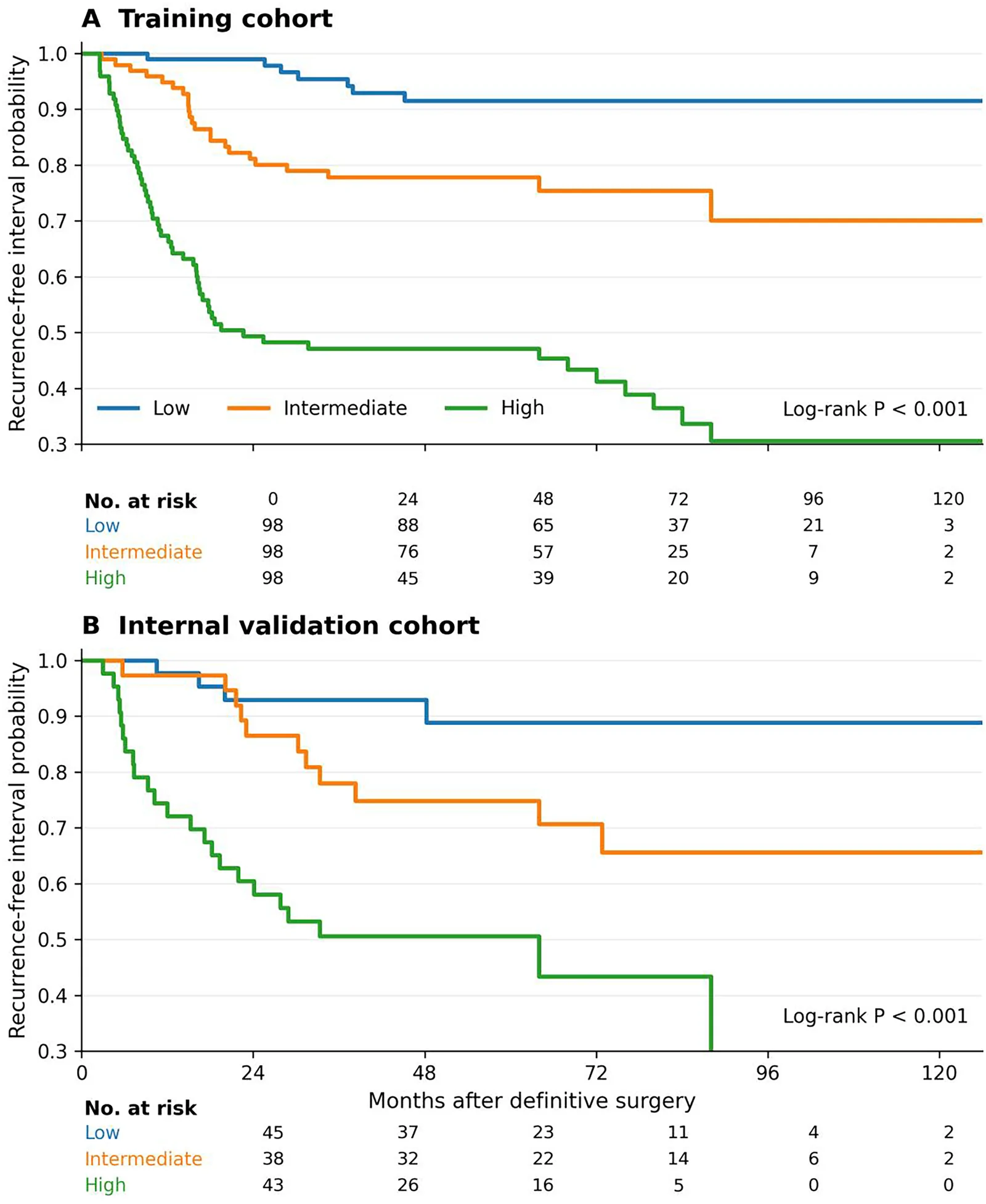
Kaplan-Meier risk stratification in training and internal validation cohorts. Kaplan-Meier RFI curves are shown for low-, intermediate-, and high-risk groups in training **(A)** and internal validation **(B)**, with numbers at risk. Cutoffs of 0.42 and 1.08 were training-derived and applied unchanged; log-rank P<0.001 in both cohorts.

**Table 6:** Risk stratification using training-derived cutoffs.

Cohort	Risk group	n	Recurrences, n (%)	5-year RFI
Training	Low	98	7 (7.1%)	91.5%
Training	Intermediate	98	23 (23.5%)	77.8%
Training	High	98	58 (59.2%)	47.1%
Validation	Low	45	4 (8.9%)	88.9%
Validation	Intermediate	38	11 (28.9%)	74.9%
Validation	High	43	23 (53.5%)	50.5%

### Model performance evaluation and comparison

Model performance is summarized in **Table 7**. The clinical model achieved C-statistics of 0.68 (95% CI 0.63-0.73) in training and 0.66 (95% CI 0.58-0.73) in validation. Corresponding values were 0.71 (0.66-0.76) and 0.70 (0.63-0.77) for the RCB-only model, 0.73 (0.68-0.78) and 0.71 (0.64-0.78) for the immune-only model, and 0.79 (0.75-0.83) and 0.75 (0.69-0.81) for the combined model. Five-year Brier scores were lowest for the combined model at 0.142 in training and 0.158 in validation. Combined-model 3-year Brier scores were 0.099 and 0.112, respectively.

**Table 7:** Predictive performance in training and internal hold-out validation cohorts.

Model	Variables	Training C-statistic	Validation C-statistic	Training 5-y Brier	Validation 5-y Brier
Clinical	cT + cN + subtype	0.68 (0.63-0.73)	0.66 (0.58-0.73)	0.181	0.194
RCB-only	RCB category	0.71 (0.66-0.76)	0.70 (0.63-0.77)	0.168	0.177
Immune-only	TILs + CD8 + FOXP3 + CD163	0.73 (0.68-0.78)	0.71 (0.64-0.78)	0.158	0.171
Combined	RCB + TNBC + CD8 + CD163	0.79 (0.75-0.83)	0.75 (0.69-0.81)	0.142	0.158

Numerical calibration was close to ideal in training and showed modest attenuation in validation. Three-year calibration slopes were 0.99 and 0.94, with intercepts of -0.01 and 0.04, in training and validation, respectively. Five-year slopes were 0.98 and 0.91, with intercepts of -0.02 and 0.06; the validation 5-year slope was 0.91 (95% CI 0.73-1.09) and the intercept 0.06 (95% CI -0.16 to 0.28).

**Figure 8** Five-year time-dependent decision curve analysis in the internal validation cohort. The combined model showed the greatest net benefit among the evaluated prediction models across threshold probabilities of approximately 15%-45%, with censoring incorporated in survival-based net-benefit estimation. Treat-all and treat-none strategies are shown for reference. These results indicate potential incremental clinical utility but do not establish treatment benefit.

**Figure 8:**
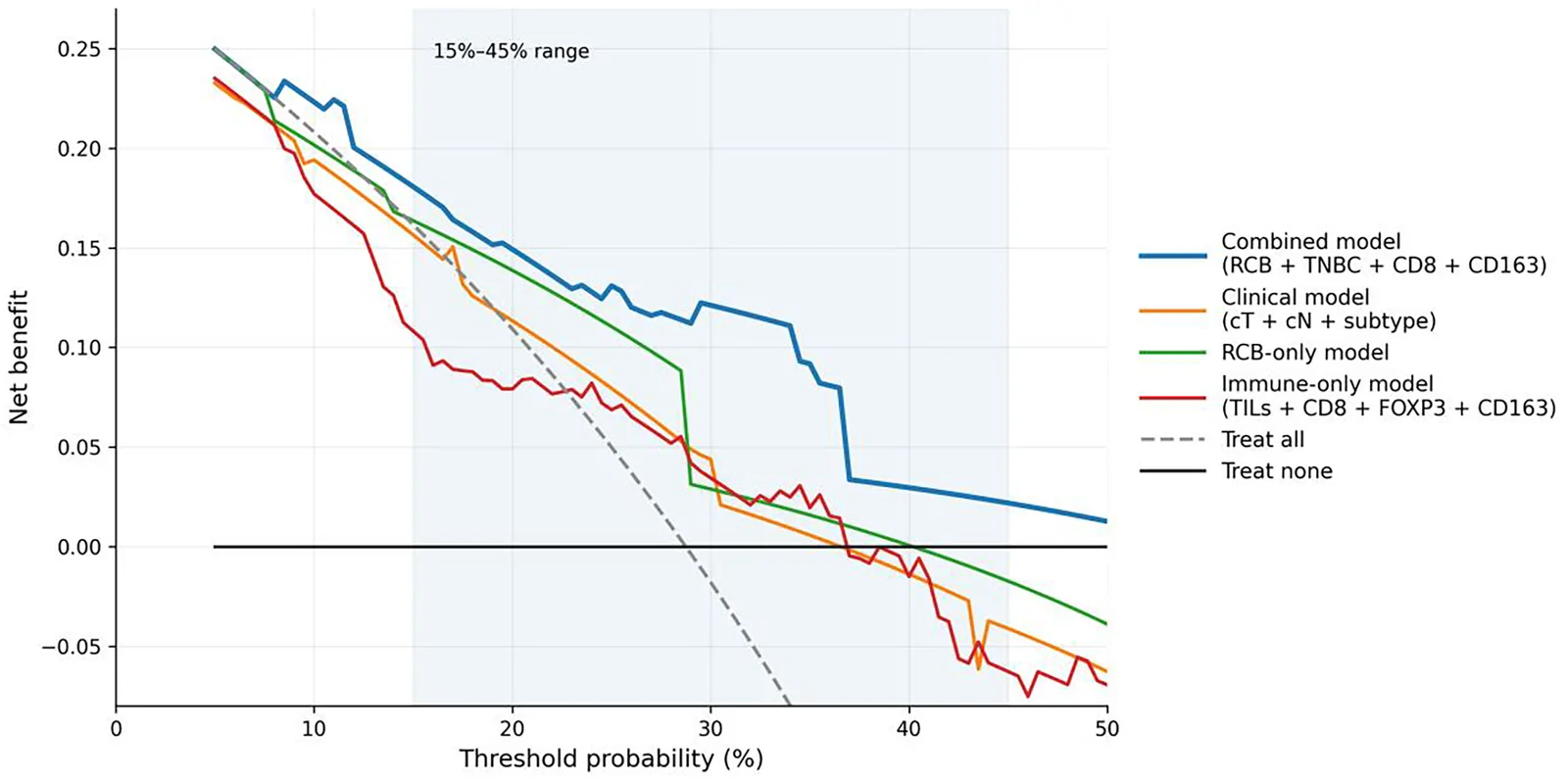
Five-year time-dependent decision curve analysis. Decision curves are shown for the combined, clinical, RCB-only, and immune-only models in the internal validation cohort together with treat-all and treat-none strategies. Censoring was incorporated using survival-based net-benefit estimation. The combined model provided the largest net benefit across threshold probabilities of approximately 15%-45%.

### Calibration, bootstrap validation, and sensitivity analyses

Validation calibration is shown in **Figure 9**. In the training cohort, the apparent combined-model C-statistic was 0.790. Repeating the complete model-development workflow in 1,000 bootstrap resamples yielded mean optimism of 0.018 and an optimism-corrected C-statistic of 0.772. The apparent and optimism-corrected 3-year Brier scores were 0.099 and 0.105, respectively; corresponding 5-year values were 0.142 and 0.151. The optimism-corrected calibration slope was 0.92. Bootstrap selection frequencies were 100% for RCB, 89% for CD8, 84% for CD163, 76% for TNBC, 34% for TILs, 28% for cN stage, and 21% for FOXP3 (**Figure 10**).

**Figure 9:**
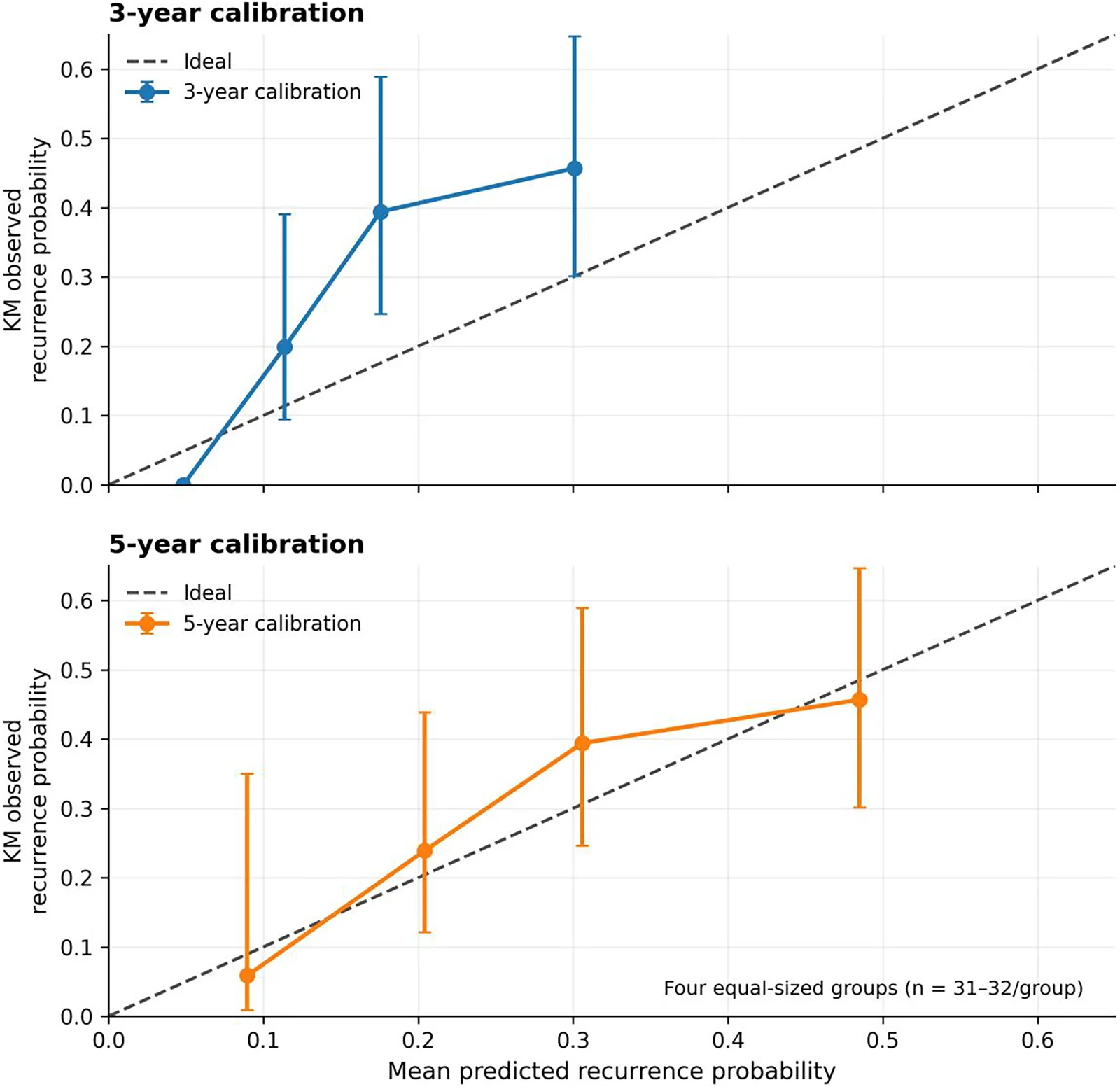
Internal hold-out validation calibration. Points show mean predicted risk versus Kaplan-Meier observed recurrence in four approximately equal-sized groups at 3 and 5 years; vertical bars are 95% confidence intervals and the dashed line indicates ideal calibration.

**Figure 10:**
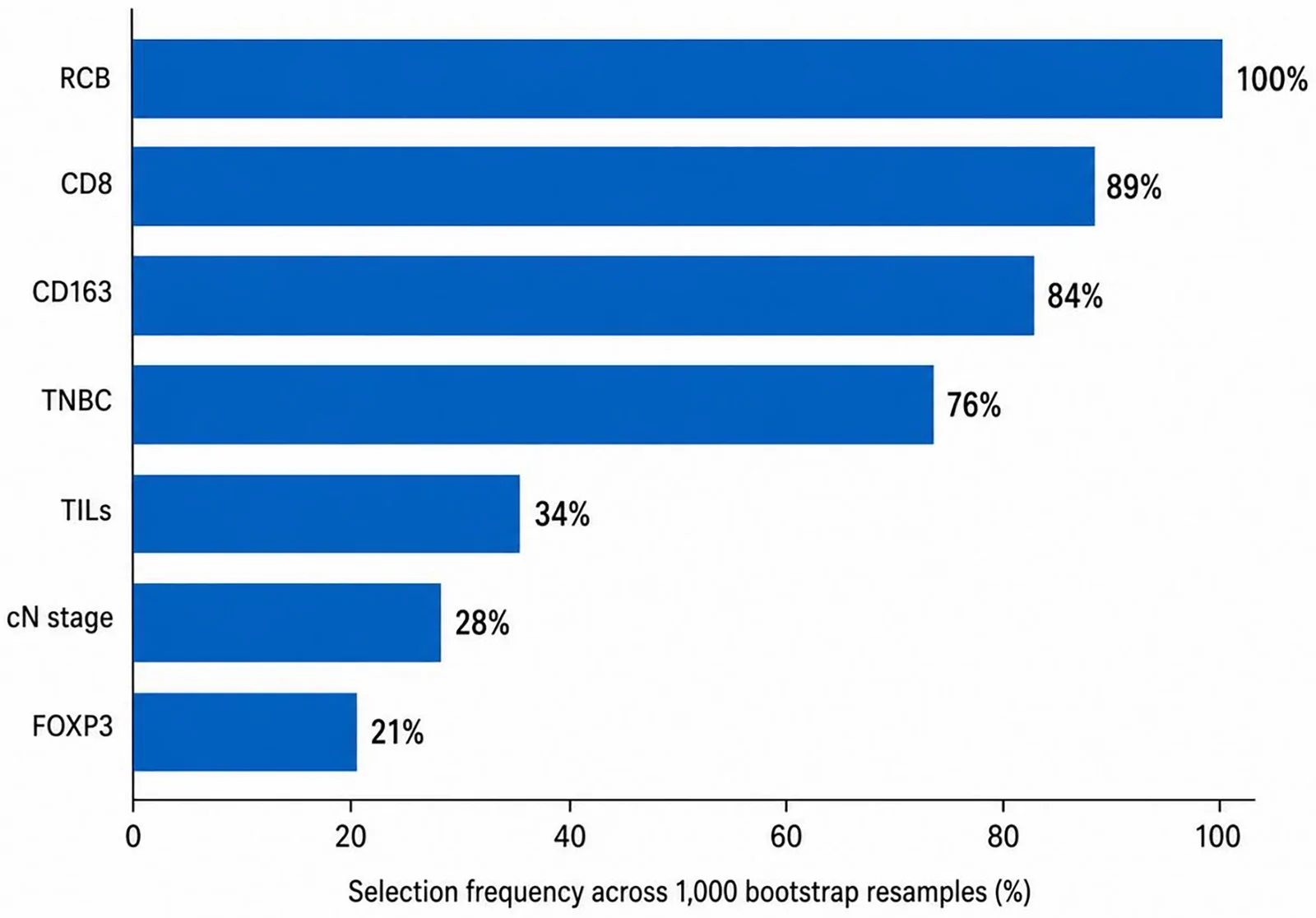
Bootstrap predictor-selection stability. Selection frequencies across 1,000 full-workflow bootstrap resamples were 100% for RCB, 89% for CD8, 84% for CD163, 76% for TNBC, 34% for TILs, 28% for cN stage, and 21% for FOXP3.

**Supplementary Figure S1**. In a treatment-adjusted sensitivity model, the combined risk score remained associated with recurrence per 1-SD increase (HR 1.82, 95% CI 1.52-2.19, P<0.001), and high versus low risk yielded an adjusted HR of 2.46 (95% CI 1.73-3.50, P<0.001); primary-predictor coefficients changed by less than 8%. Excluding RCB-0 cases left 301 non-pCR patients with 116 recurrence events and did not materially change association directions (C-statistic 0.74). Complete-case analysis of 397 patients gave a C-statistic of 0.76 and high- versus low-risk HR of 2.41 (95% CI 1.69-3.44). Counting the nine deaths without prior recurrence as events produced 135 composite RFS events (94 training, 41 validation) with materially unchanged model discrimination and effect estimates.

## Discussion

In this single-center retrospective cohort, RCB and selected post-neoadjuvant immune features provided complementary prognostic information for recurrence. The final model retained RCB category, TNBC status, CD8 status, and CD163-positive macrophage proportion and achieved C-statistics of 0.79 in training and 0.75 in internal hold-out validation. The model also showed reasonable time-specific prediction error and calibration, with an optimism-corrected C-statistic of 0.772 after full-workflow bootstrap validation. These results support incremental prognostic information beyond RCB alone while remaining preliminary in the absence of external validation.

The prognostic value of RCB has been extensively validated in multiple independent cohorts, establishing it as a continuous, reproducible metric that outperforms binary pCR classification for risk stratification (16). Our findings corroborate these reports, demonstrating progressive increases in 5-year recurrence rates from 8.4% in RCB-0 to 45.3% in RCB-III patients. Recent large-scale multicenter studies have confirmed the consistency of RCB as a prognostic indicator across diverse patient populations and treatment protocols (17). The continuous nature of the RCB index provides granular prognostic information particularly valuable for the 70-80% of patients with residual disease who would be classified simply as “non-pCR” by binary response criteria (18). However, RCB assessment alone captures only the anatomical extent of residual tumor burden without incorporating biological features that influence progression risk, motivating our investigation of complementary immune biomarkers.

TILs remain an important descriptive feature of the breast-cancer immune microenvironment, and their distribution across molecular subtypes in this cohort was consistent with prior literature (19–21). In the present prediction workflow, however, TILs were not retained in the final LASSO-selected model, and bootstrap selection frequency was 34%. This distinction is important: an immune marker may characterize post-treatment biology without providing stable incremental prognostic information once RCB and correlated immune measures are considered jointly.

CD8+ cell density showed a favorable association with recurrence, with CD8-high status retained in the final model (HR 0.64, 95% CI 0.43-0.95). CD163-positive macrophage proportion showed an adverse association (HR 1.60, 95% CI 1.08-2.37). These findings are biologically compatible with literature on effector T-cell activity and macrophage-mediated immunoregulation (22–24), but CD163 positivity should not be interpreted as proof of a fixed M2 state. The post-neoadjuvant setting also complicates biological interpretation because immune-cell density is measured after substantial therapy-induced remodeling of tumor and stromal compartments.

The immune-only clustering and PCA analyses were designed to separate the descriptive immune feature space from RCB itself. RCB was not included in either construction step; instead, RCB was overlaid after cluster and principal-component generation. The resulting association between lower RCB and a more immune-active profile therefore does not arise mechanically from inclusion of RCB in the clustering variables. The non-pCR sensitivity analysis further indicated that the direction of the final-model associations was not driven solely by the distinctive measurement context of RCB-0 tumor beds. Methodologic recommendations specifically support TIL assessment in residual disease after neoadjuvant therapy (25), and neoadjuvant HER2-positive studies have likewise shown context-dependent prognostic associations for immune infiltration (26).

The combined model improved discrimination relative to the clinical, RCB-only, and immune-only models, but the magnitude of improvement should be interpreted as prognostic rather than treatment-predictive. Postoperative therapies were strongly linked to subtype, residual burden, and calendar era; treatment-adjusted sensitivity analysis therefore served as a robustness assessment rather than as the primary etiologic model. The stability of the combined risk score after adjustment supports an association with recurrence risk but does not demonstrate that changing therapy on the basis of the score would improve outcomes. Therapy-induced remodeling and immune-regulatory mechanisms involving regulatory T cells and macrophages have been described in related literature (27–29), and the prognostic interpretation of TILs under HER2-targeted therapy is likewise treatment-context dependent (30).

Decision curve analysis suggested higher 5-year net benefit for the combined model over thresholds of approximately 15%-45%. This finding may indicate incremental clinical utility for risk communication or future stratified-study design, but it does not establish a treatment-selection rule. External validation in an independent cohort, followed by prospective assessment of clinical impact, is required before the model could be used to intensify, de-escalate, or otherwise direct postoperative therapy.

Several limitations require emphasis. The single-center retrospective design limits generalizability, and the 70:30 split represents internal hold-out validation rather than independent validation. Although full-workflow bootstrap validation reduced concern about optimism, external validation remains essential. Immune features were measured in post-treatment tissue, and RCB-0 cases required evaluation of the treated tumor-bed/stromal area rather than residual invasive tumor. Interobserver ICCs were high, but conventional immunohistochemistry cannot capture spatial or functional immune states, and archived assay metadata did not permit uniform reconstruction of antibody clone and manufacturer information for every case. CD163 was therefore interpreted as a macrophage phenotypic marker rather than a conclusive M2 marker. Cross-tumor biomarker literature similarly emphasizes assay standardization and implementation constraints (31).

Prediction-model reporting and appraisal frameworks emphasize transparent specification of model development, internal validation, calibration, and applicability (32, 33), principles reflected in the present reporting of coefficients, baseline survival, time-specific Brier scores, calibration parameters, and bootstrap optimism. Exploratory subgroup analyses remained underpowered because only 126 primary recurrence events occurred overall, including 88 in training, and no subgroup-specific treatment conclusions are warranted. The study also spans an era of changing systemic therapy and imaging practice; multimodal nodal assessment can complement pathology-based risk stratification in breast cancer (34). Future work should prioritize multicenter external validation, assay harmonization, spatial immune profiling, and prospective evaluation of incremental clinical impact.

In conclusion, a parsimonious model integrating RCB, TNBC status, CD8, and CD163-positive macrophage proportion provided incremental prognostic information for post-neoadjuvant recurrence in this retrospective cohort. Internal hold-out and bootstrap validation supported moderate-to-good discrimination with acceptable calibration, while sensitivity analyses showed consistent association directions. The model should be considered an internally validated prognostic tool for further study rather than a basis for current treatment intensification or de-escalation; independent external validation is required before clinical implementation.

## Data Availability

The original contributions presented in the study are included in the article/**Supplementary Material**. Further inquiries can be directed to the corresponding author.
