# Supplementary material for: Predicting recurrence risk after neoadjuvant therapy in breast cancer: a single-center retrospective cohort study integrating residual cancer burden and immune microenvironment features
Source: Front Oncol. 2026 Oct 14;16:1925326. doi: 10.3389/fonc.2026.1925326 (PMC13713326; doi:10.3389/fonc.2026.1925326)
Supplement: Supplementary file 1 [file DataSheet1.docx]

**Supplementary Material**

Predicting Recurrence Risk After Neoadjuvant Therapy in Breast Cancer: A Single-Center Retrospective Cohort Study Integrating Residual Cancer Burden and Immune Microenvironment Features

**Supplementary Table S1. Postoperative Treatment and Calendar-Era Distribution by Cohort**

| **Characteristic** | **Overall** | **Training** | **Validation** |
| --- | --- | --- | --- |
| Capecitabine | 46 (11.0%) | 32 | 14 |
| HER2-directed therapy | 143 (34.0%) | 101 | 42 |
| Endocrine therapy | 244 (58.1%) | 171 | 73 |
| Radiotherapy | 326 (77.6%) | 228 | 98 |
| Continued immunotherapy | 41 (9.8%) | 29 | 12 |
| Surgery in 2015-2019 | 181 (43.1%) | 126 | 55 |
| Surgery in 2020-June 2024 | 239 (56.9%) | 168 | 71 |

**Supplementary Table S2. Missing Candidate-Predictor Data**

| **Variable** | **Overall missing** | **Training** | **Validation** |
| --- | --- | --- | --- |
| TILs | 4 | 3 | 1 |
| CD8 | 7 | 5 | 2 |
| FOXP3 | 9 | 6 | 3 |
| CD163 | 11 | 8 | 3 |
| Patients with >=1 missing predictor | 23 (5.5%) | 16 (5.4%) | 7 (5.6%) |
| RCB | 0 | 0 | 0 |
| Outcome | 0 | 0 | 0 |

**Supplementary Table S3. Calibration and Bootstrap Internal Validation**

| **Metric** | **Training** | **Validation / corrected result** |
| --- | --- | --- |
| 3-y calibration slope | 0.99 | 0.94 |
| 3-y calibration intercept | -0.01 | 0.04 |
| 5-y calibration slope | 0.98 | 0.91 (95% CI 0.73-1.09) |
| 5-y calibration intercept | -0.02 | 0.06 (95% CI -0.16 to 0.28) |
| Apparent C-statistic | 0.790 | - |
| Mean bootstrap optimism | 0.018 | - |
| Optimism-corrected C-statistic | - | 0.772 |
| Apparent 3-y Brier | 0.099 | - |
| Optimism-corrected 3-y Brier | - | 0.105 |
| Apparent 5-y Brier | 0.142 | - |
| Optimism-corrected 5-y Brier | - | 0.151 |
| Optimism-corrected calibration slope | - | 0.92 |

**Supplementary Table S4. Exploratory Molecular-Subtype Subgroup Analysis**

| **Molecular subtype** | **n** | **Recurrences** | **High vs low risk HR (95% CI)** |
| --- | --- | --- | --- |
| Luminal A | 76 | 14 | 1.61 (0.72-3.62) |
| Luminal B | 178 | 44 | 2.08 (1.30-3.34) |
| HER2-enriched | 92 | 27 | 2.24 (1.21-4.16) |
| TNBC | 74 | 41 | 2.52 (1.45-4.38) |
| Total | 420 | 126 | P for interaction = 0.67 |

**Supplementary Table S5. Sensitivity Analyses**

| **Analysis** | **Population / events** | **Result** |
| --- | --- | --- |
| Treatment-adjusted combined score | Full cohort | HR per 1-SD 1.82 (95% CI 1.52-2.19), P<0.001 |
| Treatment-adjusted high vs low risk | Full cohort | HR 2.46 (95% CI 1.73-3.50), P<0.001; primary coefficients changed <8% |
| Non-pCR analysis | n=301; 116 recurrences | RCB-II vs I HR 1.71; RCB-III vs I HR 3.19; TNBC HR 1.42; CD8-high HR 0.68; CD163-high HR 1.51; C-statistic 0.74 |
| Complete-case analysis | n=397 | C-statistic 0.76; high vs low HR 2.41 (95% CI 1.69-3.44) |
| Composite RFS sensitivity | 420; 135 events (94 training, 41 validation) | Model discrimination and effect estimates materially unchanged |

**Supplementary Table S6. Five-Year Time-Dependent Decision Curve Net Benefit in the Internal Validation Cohort**

| **Threshold** | **Treat none** | **Treat all** | **Clinical** | **RCB-only** | **Immune-only** | **Combined** |
| --- | --- | --- | --- | --- | --- | --- |
| 15% | 0 | 0.162 | 0.156 | 0.164 | 0.108 | 0.181 |
| 20% | 0 | 0.109 | 0.113 | 0.139 | 0.079 | 0.149 |
| 25% | 0 | 0.050 | 0.079 | 0.110 | 0.069 | 0.131 |
| 30% | 0 | -0.018 | 0.044 | 0.029 | 0.034 | 0.121 |
| 35% | 0 | -0.096 | 0.006 | 0.016 | 0.019 | 0.092 |
| 40% | 0 | -0.188 | -0.014 | 0.001 | -0.015 | 0.030 |
| 45% | 0 | -0.296 | -0.041 | -0.017 | -0.063 | 0.022 |

**Supplementary Figure S1. Exploratory Molecular-Subtype Subgroup Analysis**

The forest plot shows high- versus low-risk hazard ratios using the training-derived risk thresholds applied unchanged across molecular subtypes. P for interaction=0.67. Given limited events within individual subgroups, these estimates are exploratory.
